# Fixation of unstable distal radius fractures by using expandable Intramedullary nailing system in adult patients

**DOI:** 10.12669/pjms.341.14239

**Published:** 2018

**Authors:** Murat Calbiyik

**Affiliations:** Murat Calbiyik, Department of Orthopedics and Traumatology, Hitit University, Faculty of Medicine, Corum, Turkey

**Keywords:** Distal radius fracture, Internal fixation, Intramedullary nailing

## Abstract

**Objective:**

To present our experience on intramedullary nailing device Sonoma Wrx (Sonoma Orthopedic Products Inc., Santa Rosa, CA, USA) used for internal fixation of extra-articular or simple intra-articular distal radius fractures in adult population.

**Methods:**

This study was conducted from February 2011 to October 2016. A total of 48 patients (mean age 47.3±5.6 years, 35.4% females) with distal radius fracture, who underwent intramedullary distal radius fixation by using Sonoma Wrx were included in this retrospective study. Clinical outcome measures (range of motion [ROM], visual analog scale [VAS]), functional outcomes (Disabilities of the Arm, Shoulder and Hand [DASH] score and Gartland-Werley score), radiographic scores (Stewart score) and parameters (radial inclination, volar tilt, radial height, radio-ulnar variance) and complications were evaluated.

**Results:**

The total surgery time was 24.3±2.3 minutes. Patients were followed up for 24.7±3.4 weeks. Complete fracture union was obtained at 5.5±0.9 weeks. The postoperative low VAS pain score (1.6±0.93) and high ROM values (76.7° for extension, 78.5° for supination, 80.1° for flexion, and 82.3° for pronation) indicated a very good clinical outcome. DASH score of 8.3±1.5 and Gartland-Werley score of 2.8±4.1 showed good functional outcome. The radiographic Stewart score was 1.0±1.2. Radial inclination, volar tilt, and radial height significantly increased (p<0.001), and radio-ulnar variance decreased (p=0.001) with surgery. No postoperative complication was recorded in 40 patients (83.3%).

**Conclusions:**

Sonoma Wrx, which is an expansible intramedullary elastic locking distal radius nail, offers a good alternative technique for internal fixation of unstable distal radius fractures with the advantage of minimum soft-tissue dissection and related postoperative complications.

## INTRODUCTION

Distal radius fractures are among the most common injuries accounting for 15-25% of all fractures.^1^ Pediatric and elderly populations are at greatest risk for this injury, which has an increasing prevalence due to increase in sports related activities and aging population.^2^,^3^

Although distal radius fracture is a common entity, there are many controversies on its diagnosis, classification, treatment, and evaluation of outcomes.^4^,^5^ Various surgical techniques for fixation of extra- and intra-articular distal radius fractures have been described without consensus on the optimal technique. The most common of these techniques are Kirschner wire (K-wire) fixation, volar plating, and intramedullary nailing.^6^

Intramedullary nailing has been introduced as a less invasive technique for internal fixation of both intra- and extra-articular distal radius fractures and for correction of post-traumatic deformity.^7^-^9^ With the advantages of minimum soft-tissue dissection, intramedullary nailing promotes effective fracture stabilization and healing.^10^ Clinical studies showed that in comparison to volar plating, intramedullary nailing provides better or comparable early postoperative functional outcomes and reduce the incidence of carpal tunnel syndrome, a major complication of distal radius fracture.^10^-^12^

Several devices for intramedullary nailing have been on the market, which showed promising outcomes for internal fixation of distal radius fractures.^9^,^13^,^14^ However, there are limited studies reporting the outcome of intramedullary nailing device Sonoma Wrx (Sonoma Orthopedic Products Inc., Santa Rosa, CA, USA), which has an expandable shaft that becomes rigid once implanted.^15^

In this study, we aimed to present our experience on clinical, functional and radiological outcomes of internal fixation of extra-articular or simple intra-articular distal radius fractures by using intramedullary nailing device Sonoma Wrx in adult population aged over 40 years.

## METHODS

A total of 48 patients (mean age 47.3±5.6 years, 35.4% females) with distal radius fracture, who underwent intramedullary distal radius fixation by using intramedullary device Sonoma Wrx in our clinic between February 2011 and October 2016 were included in this retrospective study. The inclusion criteria were unstable extra-articular or simple intra-articular distal radius fractures suitable for close reduction (Arbeitsgemeinschat fur Osteosynthesesfragen [AO] types A2.1, A2.2, A2.3, A3.1, C2.1). Patients with open or contaminated wounds without adequate soft tissue coverage, open physes, significantly displaced intra-articular fragments, irreducible articular or extra-articular fractures, partial articular fractures involving the volar or dorsal rim (AO type 23-B) and cases in which the articular fragments are small, comminuted, and cannot be reduced adequately by closed or percutaneous means and those with fracture extension proximally into the metaphyseal-diaphyseal bone were excluded from the study.

The study was conducted in full accordance with the Helsinki Declaration and local guidelines and legislations, and the permission was obtained from our institutional ethics committee for the use of patient data for publication purposes.

### Surgical technique

Initially, closed reduction and circular casting were applied in all the patients with extra-articular or simple intra-articular fractures of the distal radius. Cases with subsequent findings on radiography following casting including volar tilt >20°, articular incongruity >2 mm, radial inclination >15°, and radial shortening >5 mm were treated surgically by using the Sonoma WRx, an expansible elastic locking distal radius intramedullary nail.

Sonoma WRx was performed under local or general anesthesia and fluoroscopic control at supine position. First, closed reduction and temporary fixation using a K-wire were performed. Subsequently, an incision two to three cm in length over the radial styloid process was made. Care was taken not to harm the superficial branches of the radial nerve. Deep dissection was performed between the synovial sheaths of the extensor carpi radialis muscle and the combined synovial sheaths of the abductor pollicis longus and extensor pollicis brevis muscles to reach 3-4 mm proximal to the radioscaphoid joint. From this point with creation of a cortical window and use of appropriate guiding system for the distal buttress screws, three divergent screws were easily placed into the distal radius and the distal fracture fragment was firmly attached with fixed angle support.

### Pre- and postoperative assessments

**Fig. 1:**
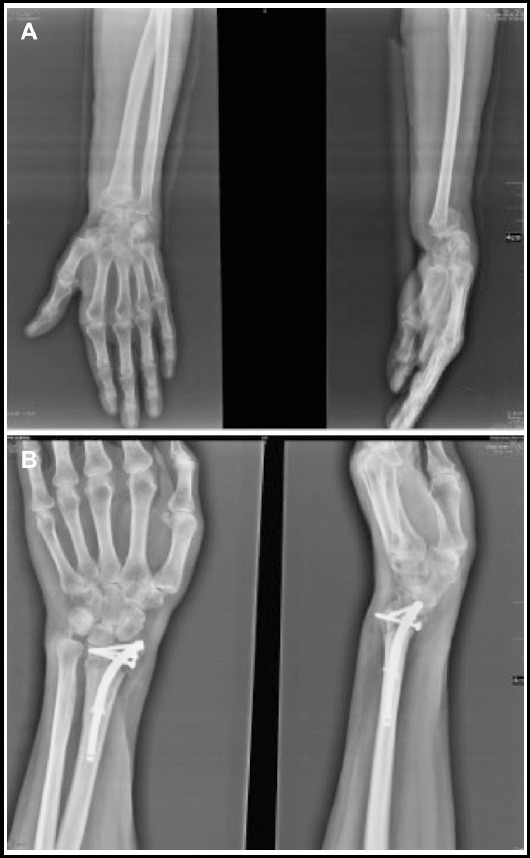
Anteroposterior and lateral radiographics of distal radius fractures before (a) and after (b) internal fixation with intramedullary nailing device Sonoma Wrx.

Data on patient demographics (age, gender), handedness, type of injury, type of distal radius fracture, concomitant fractures, duration of follow-up, length of hospital stay, total surgery and scopy time, and time to fracture healing were recorded in each patient. Clinical outcome measures included range of motion (ROM), visual analog scale (VAS), functional outcomes (patient reported Disabilities of the Arm, Shoulder and Hand [DASH] score and clinician-based Gartland-Werley score)^16^, radiographic scores (Stewart score)^17^ and parameters (radial inclination, volar tilt, radial height, radio-ulnar variance) related to quality of radiographic reduction and complications. Radiographic criteria of acceptable healing defined by Graham^18^ were used for evaluation. Pre- and postoperative radiographic images of a patient were given in Fig.1.

### Statistical analysis

Data were expressed as mean ± standard deviation (SD) or percent (%) where appropriate, and evaluated by Shapiro-Wilk's test for normality of distribution. Chi-square or Fisher's exact test was used for the comparison of categorical data, while the Student's t test or Mann-Whitney U test was used to compare continuous data for normally or non-normally distributed data, respectively. To compare preoperative and postoperative continuous variables, the paired t test or Wilcoxon signed rank test was used. Statistical analysis was made using IBM SPSS Statistics for Windows, Version 22.0 (IBM Corp., Armonk, NY, USA). p<0.05 was considered statistically significant.

## RESULTS

The distal radius fracture was caused by fall (56.2%), vehicle accident (22.9%), or sports injury (16.7%). The type of fracture was almost equally distributed between A2.1, A2.2, A2.3, A3.1, C2.1 types. The majority of patients (79.2%) had no concomittant fractures. The basic demographic and clinical characteristics of patients were summarized in Table-I.

**Table-I:** Baseline clinical characteristics, operational and outcome measures of study patients.

Study population (n=48)		
Age (year), mean ± SD		47.3±5.6
Gender	Male	31 (64.6)
	Female	17 (35.4)
Handedness	Right	22 (45.8)
	Left	26 (54.2)
Type of injury	Fall	27(56.2)
	Vehicle accident	11(22.9)
	Sports injury	8(16.7)
	Assault injury	2 (4.2)
Type of distal radius fracture	A2.1	14 (29.1)
	A2.2	9 (18.8)
	A2.3	12 (25)
	A3.1	7 (14.6)
	C2.1	6 (12.5)
Concomitant fractures	None	38 (79.16)
	Calcaneus fracture	2 (4.2)
	Humerus fracture	1 (2.1)
	Lumbar vertebral fracture	1 (2.1)
	Femur fracture	2 (4.2)
	Malleolus fracture	1 (2.1)
	Costa fracture	1 (2.1)
	Clavicula fracture	1 (2.1)
Total surgery time (min)		24.3±2.3
Scopy time (min)		14.9±2.6
Duration of follow-up (week)		24.7±3.4
Length of hospital stay (day)		3.4±0.86
Time to fracture union (week)		5.5±1
VAS pain score		1.6±0.93
ROM (°)	Flexion	80.1±6.2
	Extension	76.7±7.8
	Pronation	82.3±3.2
	Supination	78.5±3.2
DASH score		8.28 ±1.49
Gartland-Werley score		2.8±4.12
Stewart score		1.0±1.2

Data are given as n (%) or mean±SD. VAS: visual analog scale, DASH: Disabilities of the Arm, Shoulder and Hand, ROM: range of motion.

The total surgery time and scopy time was 24.3±2.3 minutes and 14.9±2.6 minutes, respectively. Following the operation, patients were followed up for 24.7±3.4 weeks on average. Complete fracture union was obtained at 5.5±0.9 weeks (Table-II).

**Table-II:** Radiographic outcome (n=48).

	Preoperative	Postoperative	p value
Radial inclination (°)	11.3±6.7	19.1±4.1	<0.001
Volar tilt (°)	-14.7±9.8	8.1±4.2	<0.001
Radial height (mm)	4.1±2.2	5.7±2.45	<0.001
Radio-ulnar variance (mm)	1.92±1.5	0.9±0.875	0.001

**Fig. 2:**
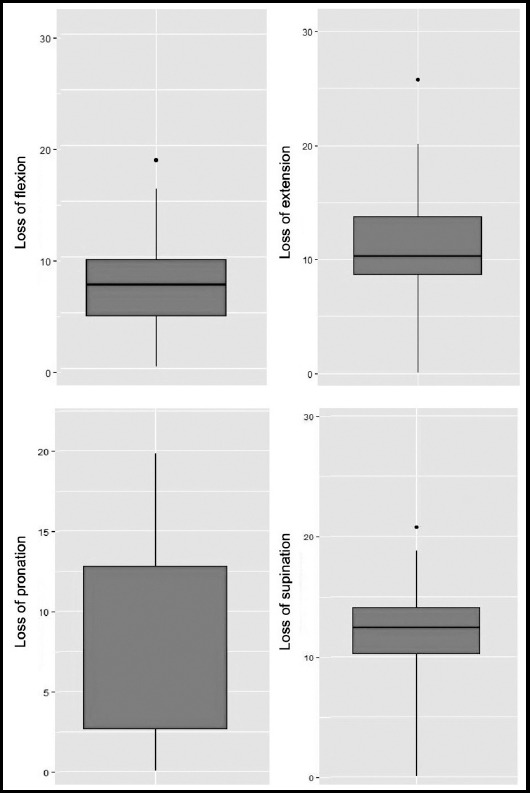
The box-and-whisker plots showing the loss in ROM degrees compared to healthy side for flexion, extension, pronation, and supination. The horizontal line within the box indicates the median, boundaries of the box indicate the 25th and 75th percentile, and the whiskers indicate the highest and lowest values of the results. The mild outliers are marked with dots.

The postoperative low VAS pain score (1.6±0.9) and high ROM values (76.7° for extension, 78.5° for supination, 80.1° for flexion, and 82.3° for pronation) indicated a very good clinical outcome of surgery (Table-I). In comparison to healthy side, the median loss in ROM was less than 15° for flexion, extension, pronation, and supination (Fig.2). The patient-evaluated DASH score of 8.3±1.5 and clinican-evaluated Gartland-Werley score of 2.8±4.1 also showed good functional outcome (Table-I).

The radiographic Stewart score was 1.0±1.2. Radiographic evaluation revealed that radial inclination, volar tilt and radial height significantly increased (p<0.001), and radio-ulnar variance decreased (p=0.001) with surgery (Table-II). The radiographic healing criteria defined by Graham indicating quality of reduction (1997) was obtained in all patients for volar tilt and articular incongruity and in 72% of patients for radial inclination.

No postoperative complication was recorded in 40 patients (83.3%). Tenosynovitis was detected in two patients; as tendon rupture, carpal tunnel syndrome, sudeck atrophy, and radial nerve paresthesia was detected in one patient each.

## DISCUSSION

In this retrospective case-series, we primarily showed that expansible intramedullary nailing device Sonoma Wrx provides a minimally invasive internal fixation with excellent functional, clinical, and radiological outcomes in extra-articular or simple intra-articular distal radius fractures in adult population.

Since distal radius fracture is a debilitating injury that causes a significant burden to the individuals and society, it should be treated effectively and safely in the shortest time after the injury to obtain best outcome.^19^ Distal radius fractures can usually be treated by closed reduction and non-invasive fixation in most of the cases.^20^ However, for extra-articular or minimally displaced intra-articular fractures that are not stable with closed reduction and external fixation, surgical fixation should be performed. Likewise, in the present study, we initially applied closed reduction and circular casting in all the patients. Cases with subsequent radiographic findings of unstable distal radius fractures were treated surgically by using the Sonoma WRx.

Several internal fixation techniques have been suggested in literature among which intramedullary nailing recently emerged as having better or equal outcome with the advantage of minimum soft-tissue dissection and short operation time compared to standard internal fixation methods like volar plating.^10^,^21^

Sonoma WRx is an expansible elastic locking distal radius nail used for a minimally invasive intramedullary fixation for extra-articular or minimally displaced intra-articular fractures that are not stable with closed reduction alone or for which prolonged immobilization is not desirable to the patient with two-part dislocated extra-articular fractures and displaced intra-articular fractures. Sonoma WRx provides fracture stability and early improvement in ROM without risking hardware irritation of surrounding structures.15 Since Sonoma Wrx is a less invasive technique, its usage may be preferable to prevent complications related soft tissue dissection.

Although various types of intramedullary nailling devices, such as Targon DR and Micro nail have been used for fixation of unstable fractures of the radius and reported to be associated with satisfactory results.^8^,^9^,^22^,^23^ experience and studies on Sonoma Wrx are limited.^24^,^25^ In our previous prospective randomized pilot study, we compared standard volar locking plate with intramedullary fixation using the Sonoma Wrx, and found that clinical and radiological outcome of two techniques were comparable and satisfactory in treatment of distal radius fractures.^25^ In the current case series, we present our latest experience with Sonoma Wrx in 39 patients with distal radius fractures.

In a meta-analysis of 14 studies on intramedullar fixation of distal radius fractures, Hardman et al.^21^ reported ROM for flexion 53.62°, extension 56.38°, pronation 69.10°, and supination 70.29°, radial inclination 18.12°, volar tilt 5.35°, radial height 8.98 mm, radio-ulnar variance 0.66 mm. In the present series we obtained very similar and excellent functional and radiological scores. We followed the patients for 24.7±3.4 weeks on average and obtained complete fracture union in all patients at 5.5±1 weeks. All the radiological parameters, which are radial inclination, volar tilt, radial height, and radio-ulnar variance, were significantly improved with intramedullary fixation of distal radius fractures by using Sonoma Wrx. Our radiologic findings indicate over 72% compliance to the radiographic healing criteria defined by Graham.18 VAS, DASH, Gartland-Werley and Stewart scores also indicate excellent clinical, functional, and radiological outcome similar to our previous report.^25^

In the present series, overall complication rate was 16.7 %, which is similar to 17-20% as reported in literature.^21^ Each of tendon rupture, carpal tunnel syndrome, sudeck atrophy, and radial nerve paresthesia was detected only in one patient, which shows that Sonoma Wrx causes limited soft-tissue injury.

### Limitations of this study

It is a retrospective non-comparative design and small sample size, which prevents us from reaching a definitive conclusion on the advantages and disadvantages of Sonoma Wrx in comparison to other intramedullary nailing systems. Nevertheless, this pilot study is among the first studies on Sonoma Wrx for internal fixation of distal radius fractures, and would guide further comparative clinical studies.

## CONCLUSION

In conclusion, Sonoma Wrx, which is an expansible intramedullary elastic distal radius nail, offers a good alternative technique for internal fixation of unstable distal radius fractures with advantage of minimum soft-tissue dissection and related postoperative complications. Further, large-scale comparative studies are needed to show the benefits of Sonoma Wrx over other intramedullary nailing systems for internal fixation of unstable distal radius fractures.
